# Body Image and Eating Disorder Education Chatbot, JEM, in Australia and Canada: First 6-Month Real-World Survey Evaluation

**DOI:** 10.2196/90783

**Published:** 2026-07-20

**Authors:** Gemma Sharp, Sara Marini, Emily Tam, Hao Hu

**Affiliations:** 1 Department of Neuroscience, Monash University, 99 Commercial Road, Melbourne, Victoria, 3004, Australia, 61 0421253188; 2 National Eating Disorder Information Centre, Toronto, ON, Canada

**Keywords:** eating disorder, body image, mental health, chatbot, conversational agent, artificial intelligence, digital health, psychoeducation, microintervention, evaluation, survey

## Abstract

**Background:**

Body image dissatisfaction, disordered eating, and eating disorders represent significant public health concerns; however, many affected individuals never access evidence-based support. We co-designed and developed a rule-based chatbot, JEM, which conducts conversations addressing evidence-based psychoeducation and psychotherapeutic microinterventions. We previously demonstrated the feasibility, acceptability, and preliminary satisfaction of the JEM chatbot in a research setting. However, broader satisfaction, experiences, and user-reported outcomes in real-world settings have not yet been investigated.

**Objective:**

This study aims to conduct a real-world evaluation of the JEM chatbot in Australia and Canada, the two countries that have hosted a deployment of the chatbot to date. Specifically, we aim to explore user satisfaction and experiences with the chatbot and within-session differences in user mood and body image satisfaction when completing the chatbot’s microinterventions.

**Methods:**

Respondents were users of the JEM chatbot aged 13 to 64 years who self-selected to complete a web-based overall evaluation survey (N=230; n=122 in Australia and n=108 in Canada) over a 6-month period. This evaluation survey included user demographic characteristics, satisfaction measures, and the System Usability Scale. Respondents for the within-session pre-post analyses were JEM chatbot users who chose to complete brief web-based surveys immediately before and after completing one of the chatbot’s microinterventions during the same 6-month period. Sample sizes varied across microinterventions, ranging from 75 to 276 respondents overall (Australia: n=34‐146; Canada: n=39‐130). These surveys included validated visual analog scales (VAS) measuring mood (anxiety, depression, happiness, confidence) and body image satisfaction (body size satisfaction, body shape satisfaction, physical attractiveness).

**Results:**

Demographic characteristics showed that survey respondents were commonly young adult cisgender women and nonbinary individuals across Australia and Canada. Respondent satisfaction with the chatbot was high in both countries (Australia: mean 76.1, SD 22.7; Canada: mean 78.8, SD 14.3), and the usability of the chatbot was rated as “excellent” in both countries (Australia: mean 86.5, SD 16.9; Canada: mean 89.5, SD 11.6) according to the System Usability Scale. Across completed microintervention surveys, patterns of within-session pre-post ratings were broadly similar in Australia and Canada, with effect sizes generally ranging from very small to large across VAS-measured mood and body image outcomes.

**Conclusions:**

The JEM chatbot achieved high satisfaction and usability ratings. Among respondents who completed pre-post surveys, immediate within-session differences in mood and body image ratings were observed following the completion of chatbot microinterventions. The study findings were broadly similar across Australia and Canada. These results provide evidence of user experience and within-session differences following engagement with JEM and support continued evaluation in future studies.

## Introduction

Eating disorders are characterized by disruptions in eating or feeding behaviors that negatively impact an individual’s physical and psychosocial functioning [1]. These disorders are present across ages, genders, and backgrounds, and their prevalence and impact are increasing [2,3]. Eating disorders are complex and involve a combination of biological, psychological, sociocultural, and environmental factors [2]. Body image concerns and body dissatisfaction have a significant association with the development of some eating disorders and extreme weight-loss behaviors [4]. Body image dissatisfaction and eating disorders are major public health concerns contributing to substantial economic and health burdens [5-7]; however, many affected individuals cannot access psychoeducation, early intervention, or timely support.

In response to the growing demand for accessible mental health support, digital mental health interventions have increased in popularity, including artificial intelligence (AI) chatbots. Chatbots offer advantages such as accessibility, instantaneous responses, and being free of cost to users, and they can act as a pathway to more individualized services [8,9]. They can also be helpful in overcoming barriers such as mental health stigma, which is particularly common in body image distress and eating disorders, or having a lack of services available [8,9]. An expanding body of research is supporting chatbot usage and efficacy in the mental health care sector [10-13]. However, despite the rapid growth in chatbot development, relatively few have been rigorously evaluated in real-world eating disorder or body image–specific contexts, with most evidence derived from controlled or early-stage pilot studies [14]. This limits understanding of how such tools perform under naturalistic conditions, including voluntary use, more diverse user populations, and nonresearch settings. As a result, there remains limited evidence regarding engagement, acceptability, and short-term outcomes of body image– and eating disorder–focused chatbots when deployed at scale.

To date, a small number of chatbots with eating disorder– and/or body image–focused evidence-based components have been reported in the literature, including Tessa [15], Topity [16], Alex [17], an early-stage unnamed chatbot for adolescents at risk of eating disorders [18], ED ESSI [19,20], and JEM [21] (formerly KIT), which is the focus of the current study. These systems differ in their primary aims and intervention focus, spanning prevention and early intervention (Tessa, Topity), support for individuals on treatment waitlists (ED ESSI), and early-stage or partially evaluated tools (Alex). Only Tessa, Topity, and ED ESSI have demonstrated effectiveness in randomized controlled trials (RCTs), with Tessa reducing weight and shape concerns in young women [15], Topity improving body image outcomes in adolescents [16], and ED ESSI reducing eating disorder pathology and mood-related outcomes in adolescents and adults awaiting treatment [19]. Certain components of Alex have also been evaluated in controlled settings, although the optimized system has not yet been fully tested [22]. Overall, despite promising early findings, there remains a lack of large-scale real-world evaluations of eating disorder and body image–focused chatbots that combine psychoeducation with brief evidence-based skill-building interventions.

JEM is a rule-based chatbot (ie, operating using predefined conversational pathways rather than generative AI) developed using the Google Dialogflow platform [23]. The chatbot was designed to deliver evidence-based eating disorder–focused psychoeducation alongside brief skills-based microinterventions [21]. The psychoeducation component provides structured information on eating disorders, including prevalence, types, warning signs, health effects, causes, and help-seeking, while the microinterventions focus on brief cognitive behavior therapy (CBT), acceptance and commitment therapy (ACT), and mindfulness-informed exercises targeting cognitive and emotional processes. These microinterventions include education on cognitive distortions or unhelpful thinking styles (CBT), practicing detaching from unhelpful thoughts via cognitive defusion exercises (ACT), and mindful breathing. Both the psychoeducation and microintervention components are delivered using multimodal formats, including text, audio, video, and graphics.

JEM was co-designed with young people aged 13 years and older with lived experience of body image concerns and/or eating disorders, parents/carers, and health professionals [21]. The co-design process provided iterative feedback on content, tone, and usability, which informed the refinement of both the psychoeducation and microintervention components. Overall, co-design findings supported the acceptability and feasibility of JEM and informed its final structure and delivery format [21]. The efficacy of JEM was not tested in an RCT setting before wider scale deployment within Australia because the development timing coincided with the COVID-19 pandemic when demand for support was high and many health initiatives were decided to be fast-tracked [24,25].

While it can be argued that RCTs are ideal for establishing evidence of efficacy as they offer structured environments for rigorous hypothesis testing and reduce the influence of external factors, their findings may not always be generalizable [26]. Evaluating a chatbot in a real-world setting is advantageous in this regard. Naturalistic evaluations are beneficial for understanding user experiences, engagement, and outcomes as they offer increased ecological validity by enabling more natural interactions and outcomes [27-29]. Moreover, they can involve a diverse user base, allowing for a better understanding of the chatbot’s usability and effectiveness across various demographics [28,29]. Additionally, real-world evaluations may capture more accurate long-term engagement, as users are not confined by the structured timeframe or artificial environment of an RCT, making it easier to evaluate the chatbot’s sustainability and broader impact [26,27]. However, such designs also introduce limitations including self-selection bias, lack of control conditions, and challenges in causal inference.

Real-world evaluations of mental health–specific chatbots are seemingly quite rare [30]. For instance, a study of Wysa, a mobile app for mental resilience and well-being, involved 129 voluntary users [31]. Data were collected via anonymous in-app feedback and assessment questionnaires from users across 23 time zones. High Wysa users showed significantly greater improvement in major depression symptoms with a moderate effect than low users, with 67.7% reporting the app as helpful and encouraging. The authors noted that these results aligned with prior RCTs on conversational agents for mental well-being [32,33] and emphasized that the naturalistic design offered scalable, real-time insights into engagement and effectiveness.

A single-session “mini-course” version of the Tessa chatbot, focused on eating disorders, was studied in users prompted via social media searches [34]. This mini-course improved body image (moderate effect) and motivation to change (small effect), but the study was not fully naturalistic due to researcher screening before mini-course access. When Tessa was deployed in a real-world US setting in June 2023, it shifted from rule-based responses to offering “off-script” dieting and weight-loss advice, which can be very harmful in eating disorder contexts. This issue was reported by users, not researchers, and attracted global attention [35,36]. Since 2023, Tessa has not been implemented in any public forums to the best of our knowledge. Overall, real-world evaluations of body image– or eating disorder–specific chatbots and mental health chatbots in general remain scarce.

The objective of the current study was to conduct a novel real-world evaluation of our body image and eating disorder education chatbot, JEM, in Australia and Canada, the 2 countries (representing 2 continents) that have hosted a deployment of the chatbot to date. Exploring both settings provided an opportunity to examine whether similar patterns of user experiences and outcomes were observed across different implementation contexts.

Given the exploratory nature of this real-world evaluation, no a priori directional hypotheses were specified. However, the microinterventions were grounded in CBT, ACT, and mindfulness approaches and were examined as within-session pre-post changes in self-reported mood and body image satisfaction following engagement with the microinterventions. The primary aim of the study was to examine user satisfaction and experiences and explore within-session pre-post changes in body image satisfaction and mood for the microinterventions. No specific eating disorder symptoms or behavioral outcomes were assessed. A secondary aim was to explore and describe any differences between the Australian and Canadian JEM deployments.

## Methods

### Ethical Considerations

This study was approved by the Monash University Human Research Ethics Committee (ID 26129), which encompassed research conducted in both Australia and Canada. Chatbot users were offered the opportunity to complete web-based surveys throughout their conversation with the JEM chatbot; however, the surveys were framed as completely optional, and users could ignore the survey and continue using the chatbot without consequence. When users clicked on a survey weblink, they were presented with detailed study information. Users were informed of their right to withdraw from the survey at any time without any negative impacts. After the detailed information, users were asked to consent by clicking on a button that they had read the information and consented to proceeding to the survey. For users aged 13 to 15 years, ethics-approved procedures required them to confirm that they had obtained parental or guardian consent via an online acknowledgment. Given the fully anonymous and low-risk nature of the research, this approach was deemed appropriate and proportionate by the approving committee. The lower age threshold of 13 years was selected in line with common minimum ages for digital platform access and social media use and to capture early adolescent users who may engage with online eating disorder–related content. Users aged 16 and 17, like those aged 18 and over, were considered to be sufficiently mature to consent for themselves without parent or guardian consent required according to our ethics approval. Users completed the surveys anonymously—they were not required to provide their name or any potentially identifying information at any point. No compensation was offered for completing surveys or using the chatbot.

### Study Design

This study used a naturalistic mixed methods design comprising 2 complementary data structures: (1) cross-sectional user evaluation surveys and (2) within-session pre-post microintervention assessments embedded within chatbot interactions. A minimum of 34 completed responses per microintervention was used as a pragmatic threshold intended to support estimation of moderate effect sizes [37], consistent with prior work [31,34], and the exploratory nature of this real-world design [38].

### Participants

Participants were individuals aged 13 years and older residing in Australia or Canada who engaged with the JEM chatbot from January 2023 to June 2023 in Australia or September 2024 to March 2025 in Canada. Geographic location was based on self-reported residence and deployment platform context within an anonymous web-based design. These time periods covered the first 6 months of JEM deployment in each country for optimal comparability, particularly in terms of the novelty of the chatbot service to the users in the respective countries [39]. In this 6-month period, system-level usage included 6862 total chatbot sessions and 39,184 interactions in the Australian deployment and 1332 sessions and 5370 interactions for the Canadian deployment according to Google Dialogflow analytics definitions [40], which are distinct from individual-level survey respondents analyzed in this study. Study participants were recruited from individuals already engaging with the JEM chatbot. All surveys (overall evaluation and microintervention surveys) were open surveys, and a self-selection sampling method was used, representing a convenience sample. No direct contact with potential participants was made by any members of the research team. Recruitment occurred in writing through the chatbot’s “Provide Feedback/Feedback Survey” and “Skill Survey” conversation options, which users could select voluntarily. These options led to web-based surveys hosted by Qualtrics [41]. Surveys were administered with standard Qualtrics platform-level fraud-prevention settings applied to reduce duplicate responses (eg, cookie-based controls) [42,43], although these do not fully eliminate the possibility of repeat submissions in anonymous online settings. Only completed questionnaires were analyzed. Due to the fully anonymous design of the chatbot and surveys, individual users could not be tracked across sessions or timepoints. Therefore, engagement is reported at the session level, and survey participation represents voluntary, self-selected responses from the overall user base (see Multimedia Appendix 1 for aggregate study flow). For users who initiated the overall survey, completion rates were 80.1%, and for users who initiated microintervention surveys, completion rates ranged from 70.2% to 75.0%. There were no significant differences between the Australian and Canadian chatbot deployments for these completion rates (all *Ps*>.05).

### Intervention

**Figure 1.**
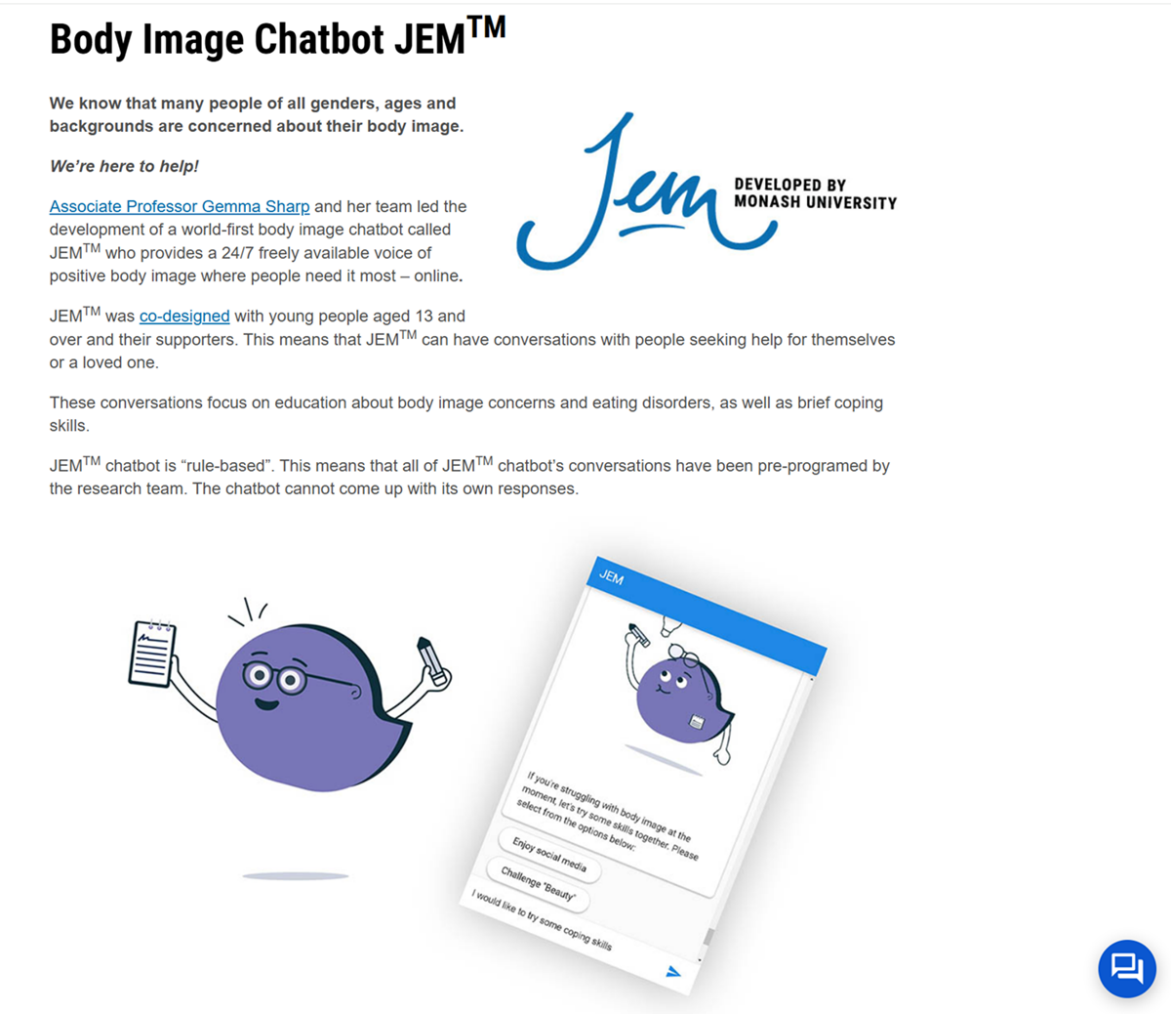
Australian version of the JEM chatbot hosted on a Monash University webpage. Users clicked on the blue speech bubble icon to start the conversation.

**Figure 2.**
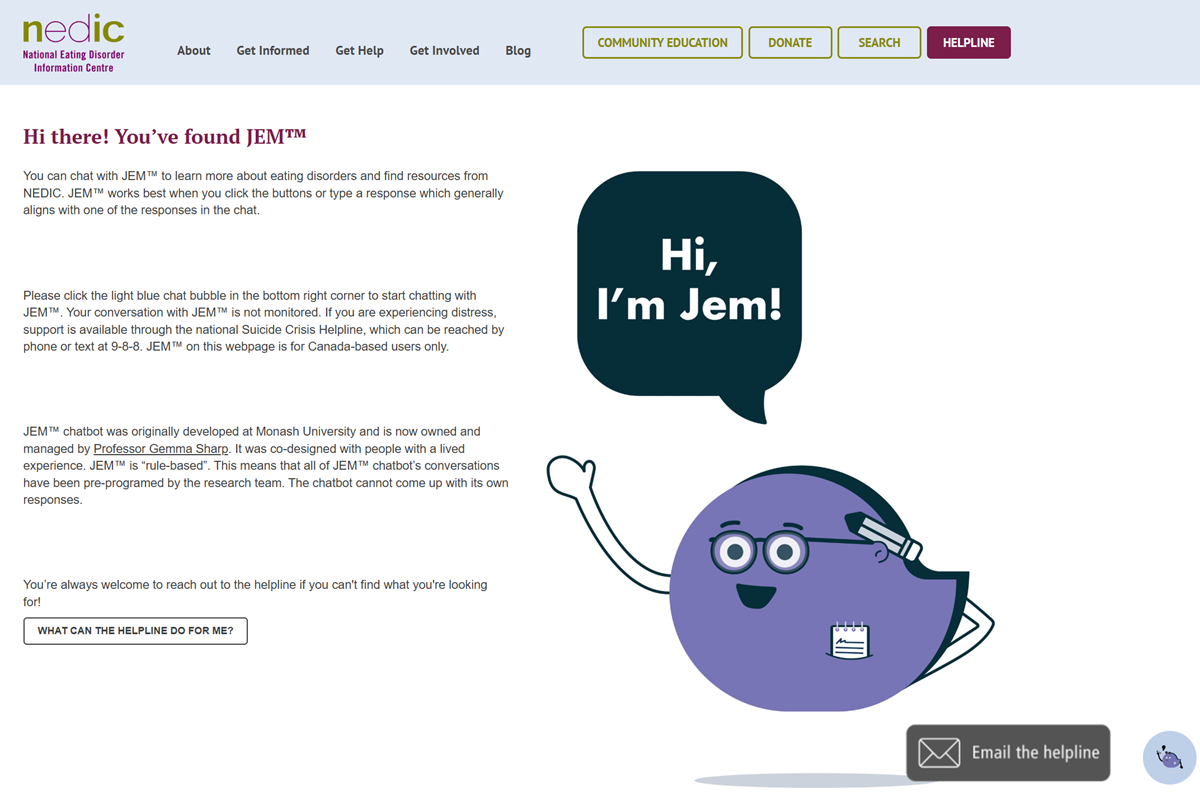
Canadian version of the JEM chatbot hosted on the National Eating Disorder Information Centre website. Users clicked on the purple JEM icon to start the conversation.

JEM was a web-based and rule-based chatbot powered by the Google Dialogflow platform [23]. The chatbot was available 24 hours per day, 7 days a week to users in Australia via a Monash University website (Figure 1) and in Canada via the National Eating Disorder Information Centre website (Figure 2). These deployment characteristics are reported to contextualize accessibility and real-world use; however, the primary focus of the intervention is the structure and content of the psychoeducational and microintervention pathways delivered within the chatbot. The chatbot was free of charge to access and users could converse with the chatbot as long as they wished to do so. There was no login or authentication required to use JEM, and usage was completely anonymous. Users were cautioned on these websites that the JEM chatbot was not monitored by any humans, and if they were experiencing an emergency, they should contact country-specific emergency services with contact details provided.

**Figure 3.**
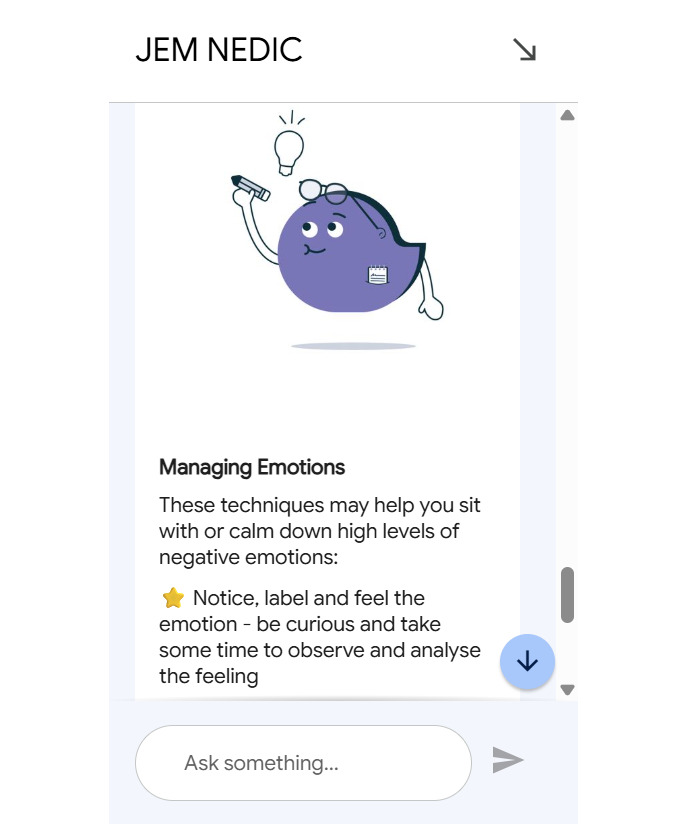
Example conversation from the JEM chatbot for the “Managing Emotions” microintervention (Canadian version).

The chatbot’s conversation content was based on evidence-based information or interventions for eating disorders, specifically psychoeducation, CBT, ACT, and mindfulness (for detailed information, see Beilharz et al [21]). Owing to the short conversational style of the JEM chatbot, the microinterventions taught by JEM were carefully selected, such as for CBT, education on cognitive distortions, for ACT, cognitive defusion exercises, and mindful breathing for mindfulness (see Figures3  4 as examples). All microinterventions were estimated to take less than 5 minutes to complete. The chatbot operated through a rule-based decision-tree structure, where user inputs were matched to predefined intents that triggered specific conversational pathways. Users engaged with JEM via either free-text inputs or menu-based options, which routed them to psychoeducation content or presented a set of available microintervention scripts. The 10 microintervention scripts were discrete, standardized conversational pathways that users could actively select from a menu of available skills. Each microintervention followed a fixed sequence of prompts and responses with no within-script branching. These scripts were delivered in multimodal formats, including text, images, audio, and video. Users self-selected into microinterventions during their interaction with the chatbot, and each script could be completed within a single session or revisited multiple times. Completion was operationalized as reaching the end of the scripted conversational pathway.

There were no major conceptual differences in intervention content and technical delivery between the Australian and Canadian deployments. In terms of safety, natural language processing was used for JEM to detect user-typed risk phrases such as “I want to kill myself,” and “I wish I was dead,” and JEM responded with 24/7 crisis support services specific to Australia or Canada. Specifically, risk detection was implemented using Google Dialogflow’s intent classification and entity extraction [44] to identify free-text inputs indicative of self-harm and suicidality. When a high-risk intent or entity was detected, the system immediately triggered a predefined response flow that bypassed standard conversational pathways and directed users to country-specific 24/7 crisis support services. There was also a “Get Urgent Help Now” option for users to select where the chatbot responded with these same crisis support services. If a user was to type a prompt that the chatbot could not match to a preprogrammed answer, the chatbot responded with “I’m just a simple bot so I’m still learning how to respond to your typed message. Try using my buttons to have a conversation with me” along with crisis support service contacts. There were no adverse events reported by users during the study period.

**Figure 4.**
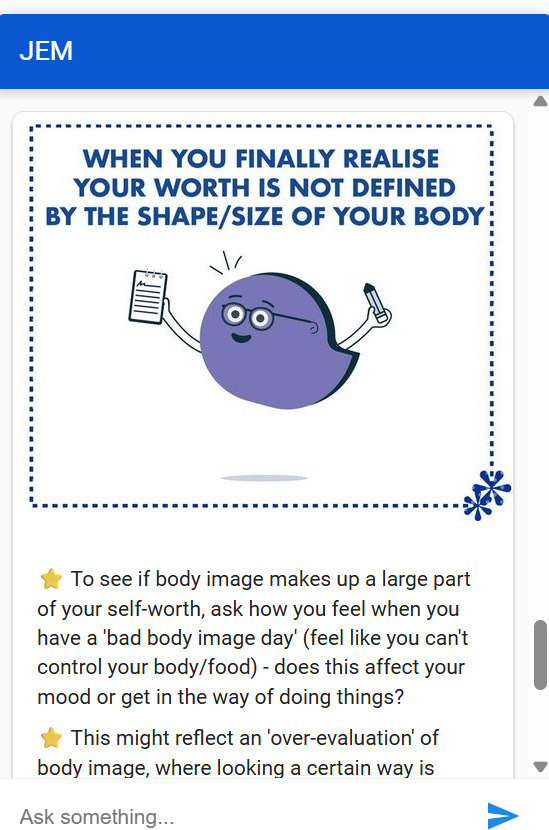
Example conversation from the JEM chatbot for the “Feeling Worthy” microintervention (Australian version).

### Measures

#### Surveys

There were 2 types of web-based surveys, hosted by Qualtrics [41], where the survey weblinks were included within the JEM chatbot’s conversation: (1) overall evaluation survey accessed via the “Provide Feedback” option and (2) skill surveys presented for every microintervention conversation (10 in total). There were no “back” options on any of the surveys for people to change their earlier responses.

#### Overall Evaluation Survey

The content and structure of this survey were based on our previous chatbot co-design research [20,21]. These items were designed as brief, study-specific indicators of user experience within an embedded chatbot context and were not intended as stand-alone psychometric instruments. In the first section of the survey, demographic characteristics were asked, particularly age, gender identity, LGBTIQA+ community membership (lesbian, gay, bisexual, transgender, intersex, queer, asexual people, or people otherwise diverse in gender, sexual orientation, and/or innate variations of sex characteristics), and ethnicity. Note that ethnicity was asked in a different format in Australia compared to Canada to suit the standard ethnicity demographics of the country [45]. The second section of the survey included categorical items addressing (1) reasons for using the chatbot, (2) whether the user found the information they were looking for, and (3) whether they intended to seek further support. The third section involved a single-item user satisfaction sliding scale ranging from 0 (“not at all satisfied”) to 100 (“completely satisfied”). This satisfaction item was followed by an optional open-text question: “Please let us know your reason(s) for your satisfaction rating.” The fourth and final section of the survey used the validated System Usability Scale (SUS) [46], which comprised 10 items that reflected various statements related to system usability (eg, “I thought the system was easy to use”). Responses were recorded on a 5-point Likert scale ranging from 1 (“strongly disagree”) to 5 (“strongly agree”). The SUS score yielded a single number between 0 and 100, with higher scores representing higher perceived usability of the system. The SUS demonstrated high reliability in this study’s sample (Cronbach *α*=0.89 for both Australian and Canadian samples).

#### Microintervention Survey

For every microintervention included in the JEM chatbot (10 in total, see Beilharz et al [21] for comprehensive descriptions), there was a web-based survey offered. If the user decided to complete the survey, they were asked for their current mood ratings for how happy, confident, anxious, and depressed they felt, with each being rated on a visual analog scale ranging from 0 (“not at all”) to 100 (“very much”). The respondents were then asked to similarly rate for body image satisfaction on visual analog scales ranging from 0 (“not at all”) to 100 (“very much”), specifically physical attractiveness, body size satisfaction, and body shape satisfaction. Note that we did not ask demographic characteristics of respondents in the microintervention surveys. Single-item visual analog scales were used to assess momentary mood and body satisfaction to minimize respondent burden and enable repeated, brief assessments within a chatbot-based design. Single-item visual analog scale measures are widely used for capturing transient, state-level affective and body image experiences and have been shown to be sensitive to within-person changes over time [47-50]. Importantly, prior research indicates that single-item measures of body satisfaction are responsive to short-term changes in both observational and online intervention contexts [51-53]. Respondents were instructed to complete the microintervention and then return to the survey immediately afterward, where they were then presented with the same mood and body image satisfaction visual analog scales again. Thus, the before and after microintervention ratings were completed within the same survey for each microintervention.

### Data Analysis

The Statistical Package for Social Sciences (SPSS; version 31; IBM Corp) [54] was used for quantitative statistical analysis. Descriptive statistics were used to analyze demographic characteristics and user satisfaction and experiences. To explore if there were differences between countries, 2-tailed independent samples *t* tests were used for continuous data and Fisher’s exact tests for categorical data (2-tailed). To examine within-session pre-post microintervention changes in mood and body image, Cohen *d* was calculated as a measure of effect size [37], with 95% CIs reported. Analyses were conducted at the survey or session level due to the fully anonymous design of the chatbot, which did not allow the linkage of responses across users or timepoints. Any missing data were handled with listwise deletion, acknowledging that missingness in optional, anonymous web-based surveys may not be completely at random. Given the exploratory and hypothesis-generating nature of these analyses, no formal correction for multiple testing was applied [55]. Applying such corrections in this context may have increased the risk of type 2 error and obscured potentially meaningful signals warranting further investigation. The results were therefore interpreted cautiously, acknowledging the increased risk of type 1 error associated with multiple comparisons. The open-ended qualitative text responses addressing reasons for chatbot satisfaction ratings were analyzed using an abbreviated content analysis approach [56], given the brevity of the responses (30 words maximum). GS conducted initial coding, with HH independently reviewing and refining codes. All authors agreed on the final content interpretations.

## Results

### User Characteristics

Across all analyses, reported Ns refer to completed survey instances (survey responses). As shown in Table 1, from the overall evaluation surveys, respondents were, on average, in their early 20s, but age distribution varied substantially, particularly in the Australian sample. The respondents predominantly identified as girls or women, followed by nonbinary individuals. Around two-thirds of respondents did not identify as LGBTIQA+; however, when they did, queer was the most common identity. There were no differences between Australian and Canadian-based respondents for any of these demographic characteristics (all *P* values >.05). The majority of respondents were European or White in Canada, and around a quarter identified as culturally and linguistically diverse (CALD) in Australia. Owing to the different style of questioning required, ethnicity was not compared between countries.

**Table 1.** Respondent demographic characteristics for overall evaluation survey by country (N=230 total).

Demographic characteristic	Australia (n=122)	Canada (n=108)
Age (y), mean (SD; range)	21.0 (12.0; 13‐64)	22.8 (6.8; 14‐43)
Gender, n (%)		
Girl or woman	99 (81.1)	87 (80.6)
Boy or man	7 (5.7)	6 (5.6)
Nonbinary	14 (11.5)	12 (11.1)
Transgender woman	0 (0)	0 (0)
Transgender man	1 (0.8)	0 (0)
Prefer not to answer	1 (0.8)	3 (2.8)
Ethnicity		
Australia CALD^a^, n (%)		
No	89 (73.0)	—^b^
Yes	31 (25.4)	—
Prefer not to answer	2 (1.6)	—
Canada, n (%)		
European or White	—	81 (75.0)
African or Black	—	3 (2.8)
Latin, South or Central American	—	3 (2.8)
East Asian	—	3 (2.8)
“Mixed”	—	6 (5.6)
Prefer not to answer	—	12 (11.1)
LGBTIQA+^c^ n (%)		
No	81 (66.4)	72 (66.7)
Bisexual	4 (3.3)	6 (5.6)
Lesbian	5 (4.1)	3 (2.8)
Queer	17 (13.9)	15 (13.9)
Gay	10 (8.2)	6 (5.6)
Asexual	0 (0.0)	3 (2.8)
Prefer not to answer	5 (4.1)	3 (2.8)

a CALD: culturally and linguistically diverse.

b Ethnicity was assessed using country-specific response categories. Blank cells indicate categories that were not applicable to that country.

c LGBTIQA+: lesbian, gay, bisexual, transgender, intersex, queer, asexual people, or people otherwise diverse in gender, sexual orientation, and/or innate variations of sex characteristics.

### Experiences and Satisfaction

As indicated in Table 2 from the overall evaluation surveys, the most common reasons in both countries for using JEM chatbot were that the respondents thought they may have an eating disorder, had issues finding information, and were simply curious about the chatbot. Respondents found the information they were looking for the vast majority of the time, and the majority intended to seek or maybe seek further support after using the chatbot. The overall satisfaction rating for the JEM chatbot was high on average, and the SUS rating was in the “excellent” category for both Australia and Canada [46]. There were no country-based significant differences for any of these findings (all *P* values >.05).

For the open-ended qualitative responses to reasons for the satisfaction ratings, this question was completed by a self-selected subset of respondents (Australia n=16; Canada n=12), which did not significantly differ between countries (all *P* values>.05). Given the lower response rates, responses were analyzed in aggregate rather than by country (Table 3). The response themes were mostly positive in nature, with the most common focused on JEM chatbot providing helpful information and being easy to use.

**Table 2.** Responses to overall evaluation survey by country (N=230 total).

Measure	Australia (n=122)	Canada (n=108)
Reasons for using the chatbot, n (%)^a^		
May have an eating disorder	71 (58.2)	61 (56.5)
Trouble finding information via other sources	32 (26.2)	29 (26.9)
Curious about the chatbot	29 (23.8)	27 (25.0)
Chatbots do not judge	13 (10.7)	12 (11.1)
Not ready to speak to a real person	10 (8.2)	9 (8.3)
Find information, n (%)		
Yes	112 (91.8)	100 (92.6)
No	10 (8.2)	8 (7.4)
Seeking further support, n (%)		
Yes	79 (64.8)	77 (71.3)
Maybe	15 (12.3)	14 (13.0)
No	14 (11.5)	12 (11.1)
I already have support	14 (11.5)	5 (4.6)
Satisfaction rating, mean (SD; range)	76.1 (22.7; 20.0‐100)	78.8 (14.3; 30.0‐100)
SUS^b^ rating, mean (SD; range)	86.5 (16.9; 20.0‐100)	89.5 (11.6; 40.0‐100)

a Percentages add up to greater than 100% as respondents could choose multiple responses.

b SUS: System Usability Scale.

**Table 3.** Themes for open-ended qualitative responses for reasons for satisfaction ratings by country (N=28 total).

Theme	Category	Total, n (%)^a^	Australia, n	Canada, n
Chatbot provided helpful information	Positive	18 (64.3)	10	8
Chatbot was easy to use	Positive	16 (57.1)	10	6
JEM character was fun or cute	Positive	7 (25.0)	3	4
Prefer a chatbot over a helpline	Positive	6 (21.4)	6	0
Chatbot had a friendly tone	Positive	4 (14.3)	0	4
Difficult to find the information sought	Negative	2 (7.1)	2	0
Chatbot conversation was targeted at too young an audience	Negative	1 (3.6)	0	1

a Percentages add up to greater than 100% as qualitative responses could be classified into multiple themes.

### Changes to Mood and Body Image

Four of the 10 total microinterventions included in the JEM chatbot’s conversation yielded sufficient completed survey responses (n≥34) from either country to conduct suitable within-session pre- or postchange analyses (Table 4). These 4 microinterventions were (1) Managing unhelpful thoughts (CBT and ACT strategies), (2) Managing emotions (ACT strategies), (3) Challenging beauty (CBT strategies), and (4) Feeling worthy (CBT strategies). Sample sizes for individual microintervention analyses varied and were modest for some subgroups; therefore, the findings should be interpreted with appropriate caution.

Across microinterventions, the within-session pre-post ratings were broadly similar in Australia and Canada, with effect sizes ranging from very small to large. The Managing unhelpful thoughts microintervention showed very small or small effect sizes across most measures, with moderate effects observed for anxiety. The Managing emotions microintervention showed the largest within-session effect sizes for depression, with moderate effects observed for body size satisfaction and the remaining measures showing very small-to-small effects. The Challenging beauty microintervention showed very small-to-moderate effects across measures, with confidence and physical attractiveness showing the largest effect sizes. The Feeling worthy microintervention showed very small-to-moderate effects across measures, with confidence, physical attractiveness, body size satisfaction, and body shape satisfaction showing the largest effect sizes.

**Table 4.** Responses to mood and body image measures before and after microintervention surveys by country.

Measure	Australia			Canada		
	Before, mean (SD)	After, mean (SD)	Cohen *d* (95% CI)	Before, mean (SD)	After, mean (SD)	Cohen *d* (95% CI)
Microintervention: Managing unhelpful thoughts (n=146 for Australia and n=130 for Canada)						
Happy	28.9 (21.9)	29.4 (24.2)	0.03 (−0.14 to 0.19)	29.7 (21.7)	29.2 (23.1)	−0.03 (−0.20 to 0.14)
Confident	20.0 (20.0)	23.4 (21.8)	0.20 (0.04 to 0.37)	20.5 (19.8)	23.0 (20.7)	0.16 (−0.01 to 0.34)
Anxious	63.0 (28.7)	53.9 (30.9)	−0.45 (−0.62 to −0.23)	64.1 (28.3)	54.4 (30.4)	−0.53 (−0.70 to −0.34)
Depressed	50.4 (31.4)	45.3 (32.0)	−0.25 (−0.42 to −0.09)	51.2 (30.7)	45.7 (31.7)	−0.29 (−0.46 to −0.11)
Physically attractive	21.9 (24.5)	22.9 (24.3)	0.06 (−0.10 to 0.23)	22.1 (24.4)	22.2 (23.0)	0.00 (−0.17 to 0.17)
Satisfied body size	17.8 (24.0)	20.4 (24.6)	0.20 (0.03 to 0.37)	18.3 (24.0)	20.0 (23.2)	0.15 (−0.02 to 0.33)
Satisfied body shape	17.8 (24.5)	20.2 (26.1)	0.18 (0.01 to 0.34)	18.2 (24.6)	19.7 (25.2)	0.14 (−0.04 to 0.31)
Microintervention: Managing emotions (n=34 for Australia and n=44 for Canada)						
Happy	36.6 (21.8)	38.3 (24.8)	0.14 (−0.20 to 0.48)	35.5 (21.2)	38.9 (23.6)	0.22 (−0.09 to 0.52)
Confident	24.2 (14.8)	26.0 (19.5)	0.12 (−0.22 to 0.46)	23.6 (15.6)	27.3 (20.2)	0.22 (−0.08 to 0.53)
Anxious	65.6 (26.4)	56.2 (28.0)	−0.38 (−0.73 to −0.03)	64.5 (26.9)	53.7 (28.3)	−0.43 (−0.73 to −0.11)
Depressed	48.8 (29.9)	34.1 (24.5)	−0.78 (−1.13 to −0.38)	48.4 (29.1)	31.9 (22.8)	−0.79 (−1.12 to −0.44)
Physically attractive	22.2 (22.8)	23.5 (26.3)	0.12 (−0.22 to 0.46)	20.9 (22.5)	22.8 (25.7)	0.18 (−0.12 to 0.48)
Satisfied body size	13.1 (17.6)	17.7 (21.7)	0.43 (0.07 to 0.78)	13.1 (17.1)	18.1 (20.8)	0.51 (0.19 to 0.82)
Satisfied body shape	17.5 (21.9)	19.2 (21.8)	0.19 (−0.15 to 0.54)	18.5 (22.5)	20.1 (22.9)	0.19 (−0.11 to 0.49)
Microintervention: Challenging beauty (n=50 for Australia and n=40 for Canada)						
Happy	40.5 (30.5)	43.6 (31.4)	0.15 (−0.13 to 0.43)	38.8 (29.2)	41.5 (29.5)	0.13 (−0.19 to 0.44)
Confident	23.5 (25.9)	33.6 (29.6)	0.47 (0.18 to 0.76)	25.2 (26.4)	32.2 (29.2)	0.38 (0.06 to 0.70)
Anxious	59.4 (31.1)	47.7 (33.5)	−0.44 (−0.73 to −0.15)	60.5 (30.5)	52.9 (33.0)	−0.32 (−0.63 to 0.00)
Depressed	46.7 (33.5)	40.4 (29.1)	−0.31 (−0.59 to −0.03)	49.2 (30.6)	43.7 (29.4)	−0.25 (−0.56 to 0.07)
Physically attractive	20.4 (25.5)	29.5 (32.1)	0.44 (0.15 to 0.73)	21.0 (26.6)	28.6 (30.9)	0.42 (0.09 to 0.74)
Satisfied body size	20.6 (27.7)	27.6 (32.7)	0.33 (0.04 to 0.62)	21.2 (29.8)	26.3 (31.9)	0.26 (−0.06 to 0.58)
Satisfied body shape	23.0 (27.7)	28.3 (30.9)	0.24 (−0.05 to 0.52)	22.6 (28.7)	27.1 (29.1)	0.21 (−0.11 to 0.53)
Microintervention: Feeling worthy (n=36 for Australia and n=39 for Canada)						
Happy	28.4 (21.1)	31.9 (24.0)	0.15 (−0.18 to 0.47)	24.3 (20.0)	31.0 (25.6)	0.23 (−0.09 to 0.54)
Confident	17.4 (16.1)	28.2 (22.5)	0.46 (0.11 to 0.80)	14.5 (15.5)	28.3 (25.1)	0.50 (0.16 to 0.83)
Anxious	63.5 (27.4)	51.2 (33.0)	−0.37 (−0.71 to −0.03)	65.4 (29.3)	55.1 (34.5)	−0.27 (−0.60 to 0.05)
Depressed	61.1 (26.1)	58.4 (29.0)	−0.10 (−0.43 to 0.23)	59.5 (26.4)	58.4 (29.6)	−0.03 (−0.35 to 0.03)
Physically attractive	12.6 (16.8)	21.7 (26.0)	0.47 (0.11 to 0.81)	10.0 (11.8)	21.7 (26.9)	0.47 (0.13 to 0.80)
Satisfied body size	8.9 (12.3)	16.8 (21.4)	0.40 (0.05 to 0.72)	7.8 (11.5)	19.2 (24.7)	0.45 (0.11 to 0.78)
Satisfied body shape	10.2 (13.5)	17.4 (22.8)	0.41 (0.06 to 0.74)	11.2 (15.5)	20.7 (27.3)	0.42 (0.09 to 0.74)

## Discussion

### Principal Findings

This study provides one of the first real-world evaluations of a mental health–focused chatbot deployed at scale across 2 countries and continents, providing insight into chatbot use under naturalistic conditions. Overall, the findings indicated that the JEM chatbot was well accepted by respondents who completed surveys in both Australia and Canada, demonstrated excellent usability, and showed immediate within-session differences in mood and body image ratings. Satisfaction was high, information needs were largely met, and most survey respondents reported an intention to seek or consider further support after using the chatbot, suggesting that JEM may function as a supportive resource that could potentially facilitate consideration of further support.

Across microinterventions, patterns of within-session pre-post ratings were broadly similar in Australia and Canada, with effect sizes generally ranging from very small to large. While the magnitude of effect sizes varied across microinterventions and outcomes, the largest effects were typically observed for depression, anxiety, confidence, and body size satisfaction outcomes. These findings reflect immediate within-session pre-post changes and should not be interpreted as evidence of causal or sustained intervention effects.

In contrast, effect sizes for happiness ratings were generally very small across the microinterventions included in the analyses. This may reflect the brief nature of the interventions, which are designed to reduce distress and maladaptive cognitions rather than induce immediate positive affect, and is consistent with theoretical expectations of CBT and ACT informed strategies [57-59].

Gender identity beyond the binary was represented in the sample, with approximately 11% of respondents identifying as nonbinary. All study analyses included these respondents within the overall sample rather than stratifying by gender. Overall, JEM’s nongendered and inclusive framing of content [21] suggests potential applicability across gender identities, although future work should directly examine differential experiences among gender diverse users, particularly their responses to psychotherapeutic microinterventions.

### Comparison With Prior Work

These findings extend the very limited literature on eating disorder– and body image–specific intervention chatbots, particularly in real-world contexts. Previous RCTs have demonstrated efficacy for Tessa, Topity, and ED ESSI under controlled conditions [15,16,19], while other chatbots have been evaluated only partially or at early stages of development [17,18]. This study extends this work by demonstrating that a rule-based chatbot can be deployed with high user satisfaction and usability in naturalistic settings. Furthermore, the patterns of within-session pre-post differences observed among JEM users aligned in direction with findings reported in more controlled trials, while recognizing the differences in study design.

Compared with prior naturalistic evaluations of mental health chatbots such as Wysa [31], this study focused on a more specific population and set of outcomes. The patterns of within-session pre-post differences in anxiety, depression, and body image satisfaction observed among JEM survey respondents were mostly consistent with those reported in broader mental health chatbot evaluations. These observations are consistent with the literature suggesting that well-designed conversational agents may be associated with positive user-reported outcomes in real-world observational settings [31,60] and align with broader discussions about the promise and fragility of AI-mediated mental health care in real-world settings [61].

Importantly, the present findings contrast with concerns raised following the real-world deployment of the Tessa chatbot in 2023 in the United States, where the chatbot deviated from its rule-based design and provided dieting and weight loss advice [35]. No adverse events or harmful outputs were reported by users during the study period. JEM’s rule-based architecture and co-designed content were intended to minimize such risks; however, all safety outcomes were not assessed in this study.

The very small effects on happiness from microinterventions also align with previous digital intervention research, where reductions in negative symptoms are often more readily observed than increases in positive affect, particularly following brief, single-session interventions [59,62]. Together, these findings suggest that JEM performs in a manner consistent with existing evidence while addressing a notable gap in real-world evaluations of body image– and eating disorder–specific chatbots.

Recent RCTs of generative AI-based conversational agents further suggest both the promise and the complexity of chatbot-delivered mental health interventions, reinforcing the importance of safety-focused design and careful real-world evaluation [63]. While the vast majority of qualitative feedback in this study was positive, with respondents reporting helpfulness, ease of use, and an engaging conversational style, a minority had negative feedback. More broadly, rule-based chatbot systems are often perceived as less flexible than emerging large language model (LLM)–based approaches, which offer more naturalistic dialogue [64]. This highlights an important design trade-off between maintaining clinically safe, structured responses and meeting evolving user expectations for more adaptive and personalized conversational experiences. Future JEM iterations could explore hybrid architectures that combine rule-based safety constraints with controlled generative or retrieval-based components to enhance conversational flexibility while maintaining clinical safety and content fidelity [65].

### Limitations and Future Research

Several limitations should be acknowledged for this study. First, the absence of a control group precludes causal inference and means changes cannot be definitively attributed to the chatbot alone. Second, outcomes were assessed immediately before and after individual microinterventions, limiting conclusions about the durability of effects. The immediate pre- and postintervention design may also be susceptible to demand characteristics and expectancy effects. However, differences were not uniform across outcomes or microinterventions, suggesting variability in within-session responses across measures and intervention content. Third, sample sizes varied across microinterventions, with 4 yielding sufficient data for analysis, which may have influenced the pattern of the findings based on differential engagement. Additionally, reliance on self-report measures and voluntary survey completion introduces potential response bias and means that the results reflect only a subset of users. Due to the fully anonymous design of JEM, which did not include login, authentication, or persistent user identifiers, individuals could not be tracked across sessions or over time. As a result, it was not possible to construct a participant-level CONSORT-EHEALTH (Consolidated Standards of Reporting Trials of Electronic and Mobile Health Applications and Online Telehealth) style flow diagram [66] tracking individuals through the chatbot content or determine precisely where attrition occurred within the chatbot journey. Engagement data are therefore reported via text and diagram at the session level, limiting the assessment of repeat usage and longitudinal engagement. Furthermore, this study did not assess changes in specific eating disorder symptoms, eating behaviors, or clinical outcomes.

Future research should examine longer-term outcomes associated with repeated or sustained chatbot use, including whether these immediate within-session differences are associated with longer-term outcomes, such as eating disorder risk or engagement with formal care. Hybrid designs combining real-world deployment with comparison conditions may help balance ecological validity with internal rigor. Further investigation into engagement patterns, personalization, and differential effects across demographic subgroups is also warranted.

### Conclusions

This study demonstrated that a co-designed evidence-based chatbot, JEM, can be deployed at scale across multiple countries, achieving high usability and user satisfaction. Immediate within-session differences in mood and body image ratings were observed following the completion of the microinterventions investigated. However, further controlled research is needed, and the longer-term effects are yet to be determined. The study findings were broadly similar across Australia and Canada. These results extend the limited real-world evidence for mental health and body image– or eating disorder–specific chatbots, demonstrating that rule-based systems with co-designed content can be acceptable to users. As digital mental health interventions evolve, future research should explore how different approaches, including rule-based and generative AI chatbots, can complement each other to provide scalable, safe, and personalized support. Collectively, these findings suggest that chatbots such as JEM may represent a feasible and acceptable low-barrier resource for individuals seeking information and support related to body image and eating disorder concerns.

## Supplementary material

10.2196/90783 Multimedia Appendix 1 Aggregate study flow for the Australian (A) and Canadian (B) JEM chatbot deployments. Ten microinterventions were available within JEM, of which 4 generated sufficient completed surveys to meet the prespecified threshold for analysis.

## Abbreviations

ACT: acceptance and commitment therapy
AI: artificial intelligence
CALD: culturally and linguistically diverse
CBT: cognitive behavior therapy
CONSORT-EHEALTH: Consolidated Standards of Reporting Trials of Electronic and Mobile Health Applications and Online Telehealth
LGBTIQA+: lesbian, gay, bisexual, transgender, intersex, queer, asexual people, or people otherwise diverse in gender, sexual orientation, and/or innate variations of sex characteristics
LLM: large language model
RCT: randomized controlled trial
SUS: system usability scale
