# Supplementary figures and images for: Body Image and Eating Disorder Education Chatbot, JEM, in Australia and Canada: First 6-Month Real-World Survey Evaluation
Source: JMIR Hum Factors. 2026 Jul 20;13:e90783. doi: 10.2196/90783 (PMC13384424; doi:10.2196/90783)

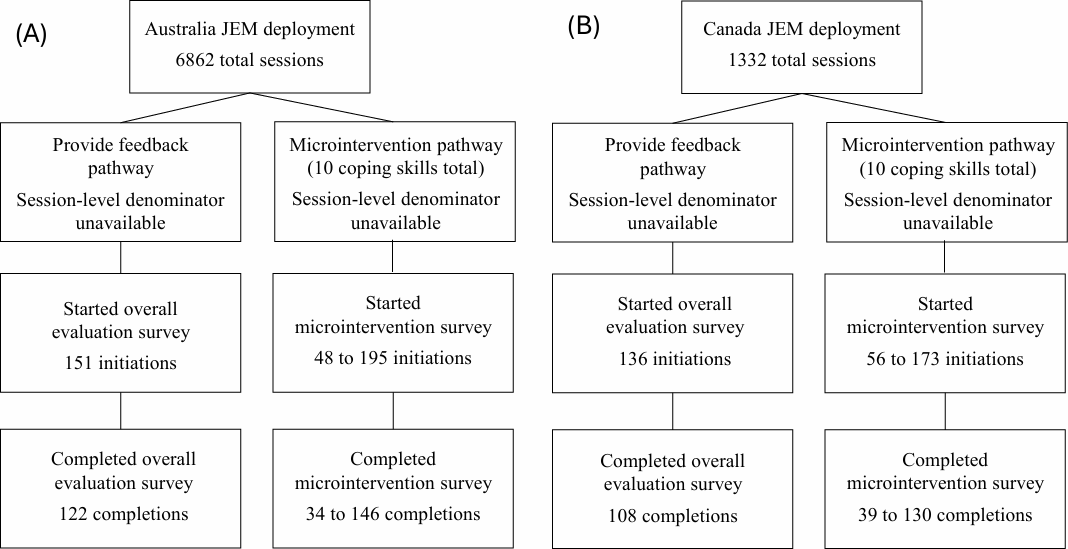

Supplement: Multimedia Appendix 1 [file humanfactors-v13-e90783-s001.png]
